# Potassium‐Ion‐Directed Three‐Dimensional Assembly of a Dense and Thermally Robust Nitrogen‐Rich Tetrazine Framework

**DOI:** 10.1002/asia.70963

**Published:** 2026-08-22

**Authors:** Jatinder Singh, Parul Saini, Richard J. Staples, Jean'ne M. Shreeve

**Affiliations:** ^1^ Department of Chemistry University of Idaho Moscow Idaho USA; ^2^ Department of Chemistry Michigan State University East Lansing Michigan USA

**Keywords:** chemistry, coordination complex, crystal, crystallography, crystal structure, hydrogen bond, ionic bonding, tetrazine, thermal stability

## Abstract

Potassium‐ion‐directed assembly of nitrogen‐rich heterocycles provides an effective approach to dense energetic salts in which coordination interactions regulate crystal packing and thermal behavior. Now we report mono‐ and dipotassium‐based salts derived from a nitrogen‐rich tetrazine framework. Single‐crystal X‐ray diffraction of dipotassium **AJE‐2** reveals extensive K–O and K–N coordination interactions that generate three‐dimensional networks reinforced by complementary N─H⋯O and N─H⋯N interactions. **AJE‐2** exhibits a room‐temperature density of 2.01 g cm^−3^, a decomposition onset of 257°C, and sensitivity toward mechanical stimuli. These findings highlight the importance of metal‐ion‐directed assembly in stabilizing nitrogen‐rich frameworks and provide insight into the design of potassium‐based energetic materials with potential relevance to primary energetics.

## Graphical abstract

A nitrogen‐rich tetrazine scaffold evolves into a highly interconnected three‐dimensional potassium framework through extensive coordination and hydrogen bonds. The resulting compound combines high density, enhanced thermal stability, and primary energetic behavior.

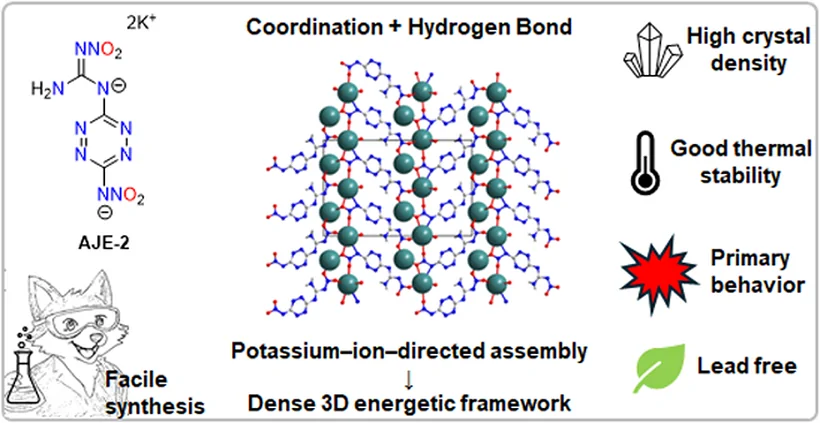

## 1 Introduction

**FIGURE 1:**
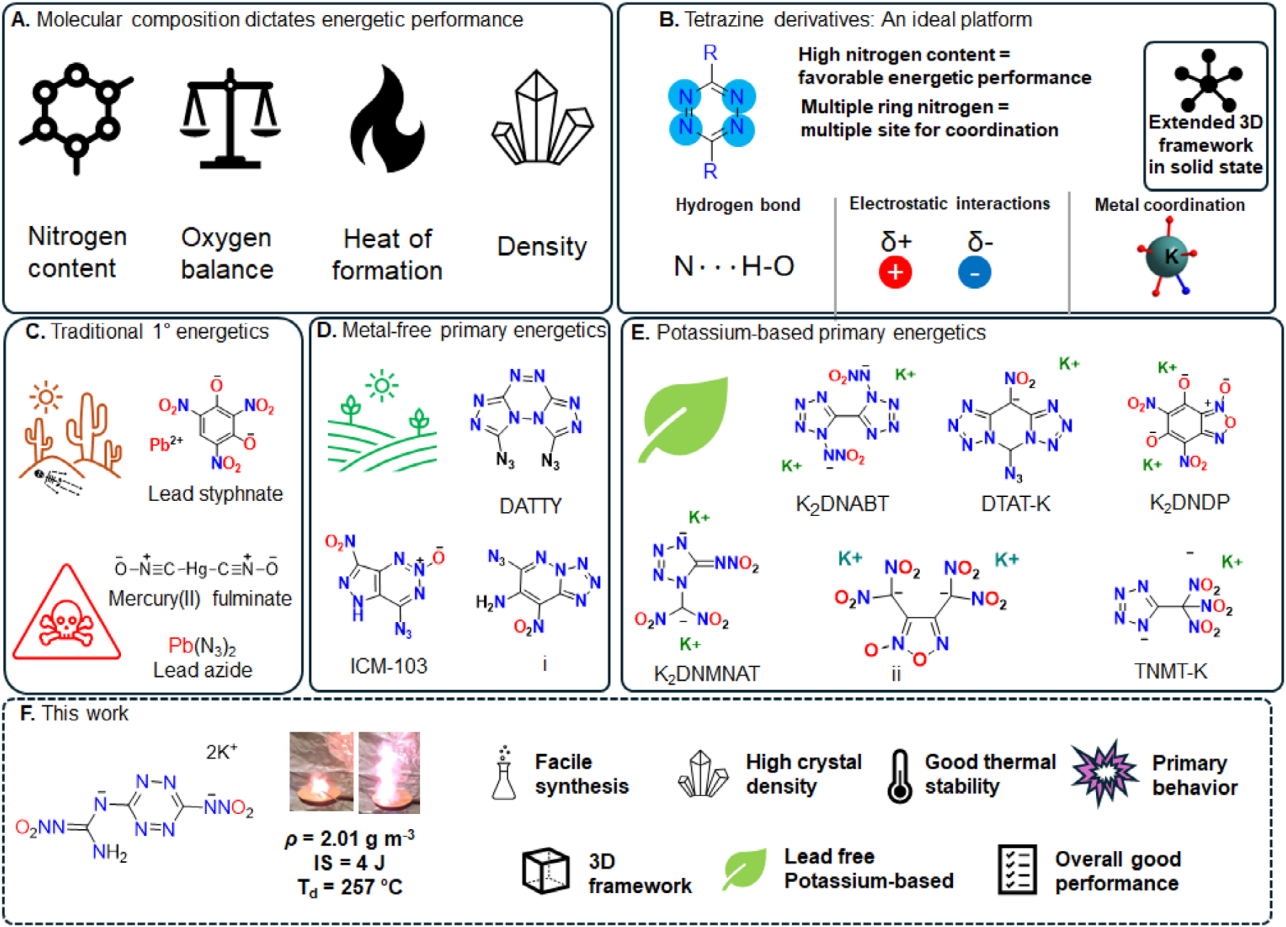
(A and B) Conceptual illustration of the relationship between molecular composition, supramolecular interactions, and material properties in tetrazine‐based energetic materials. (C) Traditional lead‐based primary explosives. (D) Representative metal‐free primary energetic materials. (E) Representative potassium‐based primary energetic materials reported in the literature. (F) Molecular structure and key characteristics of **AJE‐2**.

The performance of energetic materials is commonly discussed in terms of molecular composition, including nitrogen content, oxygen balance, density, and heat of formation (Figure 1A) [1, 2, 3, 4, 5, 6, 7, 8, 9, 10]. While these parameters are undoubtedly important, compounds possessing similar energetic functionalities often exhibit markedly different physicochemical properties [11, 12, 13, 14, 15, 16, 17, 18, 19, 20]. Such differences originate not from the molecular structures themselves but from the manner in which molecules organize within the crystal lattice [21, 22, 23, 24, 25]. Therefore, understanding how weak interactions govern crystal packing remains an important goal in the design of future energetic materials (Figure 1) [26, 27, 28, 29, 30].

Among nitrogen‐rich heterocycles, tetrazine derivatives provide an attractive platform for exploring these relationships [31, 32, 33, 34, 35]. Their high nitrogen content contributes to favorable energetic performance, while the abundance of ring nitrogen atoms offers multiple sites for intermolecular recognition [36, 37, 38, 39, 40, 41, 42]. Consequently, tetrazine‐based systems frequently assemble through a combination of hydrogen bonds, electrostatic interactions, and metal coordination which lead to extended 3D networks in the solid state (Figure 1B). These interactions strongly influence density and thermal stability, properties that are particularly important for energetic compounds. Primary energetic materials have traditionally relied on lead‐containing compounds, including lead azide, and lead styphnate (Figure 1C). More recent efforts have focused on metal‐free primary energetic materials as alternatives that reduce the environmental and health concerns associated with heavy metals (Figure 1D). Nevertheless, achieving a favorable combination of density, thermal stability, sensitivity, and reliable initiation behavior remains challenging. Potassium‐containing energetic salts represent a unique class of materials in which crystal packing is influenced not only by the energetic anion but also by the coordination preferences of the counterion (Figure 1E) [43, 44, 45, 46, 47, 48, 49, 50]. Potassium ions are especially interesting in this regard because their large ionic radius and flexible coordination environment enable simultaneous interactions with multiple oxygen and nitrogen donors. As a result, potassium ions can connect neighboring energetic building blocks into extended frameworks rather than simply balancing charge [51, 52, 53, 54, 55, 56, 57, 58, 59, 60, 61]. Despite the prevalence of potassium salts in energetic materials, the structural consequences of such coordination‐driven assembly remain comparatively underexplored.

In the course of our investigations of nitrogen‐rich tetrazine derivatives, we obtained a dipotassium salt derived from 2‐nitro‐1‐(6‐(nitroamino)‐1,2,4,5‐tetrazin‐3‐yl)guanidine. Single‐crystal X‐ray diffraction shows that the potassium ions play an active role in directing crystal growth through extensive K–O and K–N coordination interactions. These contacts generate a 3D framework that is further reinforced by complementary N─H⋯O and N─H⋯N hydrogen bond interactions. The resulting architecture illustrates how coordination bonding and hydrogen bonding can operate cooperatively to produce a densely connected nitrogen‐rich lattice. This study highlights the importance of the coordination framework in energetic materials and provides insight into the structural role of alkali metal ions within nitrogen‐rich energetic systems.

## 2 Results and Discussions

**SCHEME 1:**
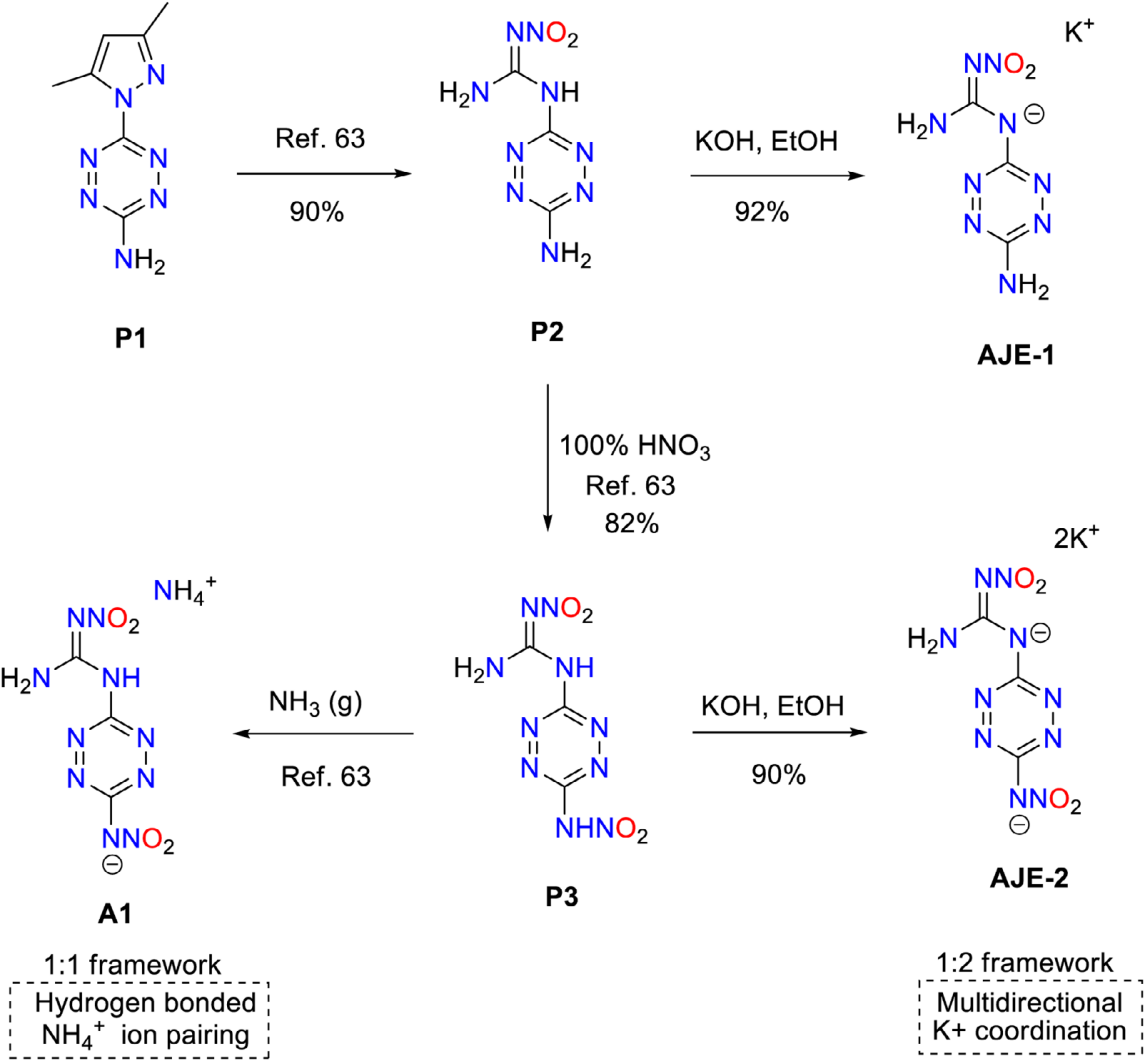
Synthesis of the monopotassium salt **AJE‐1** and dipotassium salt **AJE‐2**.

The synthetic routes to **AJE‐1** and **AJE‐2**, together with the counterion‐dependent assembly behavior of the nitrated intermediate P3, are outlined in Scheme 1. The known tetrazine precursor P1 was converted to 1‐(6‐amino‐1,2,4,5‐tetrazin‐3‐yl)‐2‐nitroguanidine (P2) according to the reported procedure [62, 63]. Treatment of P2 with KOH in ethanol resulted in selective deprotonation of the nitroguanidine moiety and formation of the monopotassium salt **AJE‐1**. Subsequent nitration of P2 with 100% nitric acid afforded P3, in which introduction of the second nitramino functionality provides an additional acidic site.

The assembly behavior of P3 is strongly dependent on the nature of the cation. Treatment with ammonia preferentially affords a monoammonium salt with a 1:1 framework‐to‐cation ratio, in which assembly is governed primarily by weak N─H⋯O/N hydrogen bonds. In contrast, treatment of P3 with two equivalents of KOH produces the doubly deprotonated dipotassium salt **AJE‐2** with a 1:2 framework‐to‐potassium ion ratio. Unlike the ammonium ion, which functions mainly as a hydrogen‐bond donor and charge‐balancing ion, potassium ions can simultaneously coordinate to multiple oxygen and nitrogen donor atoms. Incorporation of two potassium ions therefore does more than compensate for the dianionic charge. Also, it increases the number of metal‐mediated connections between neighboring anions and enables propagation of the coordination network in multiple directions.

Thus, nitration followed by double deprotonation converts the predominantly hydrogen‐bonded 1:1 ammonium assembly into a highly connected 1:2 dipotassium coordination framework. This comparison highlights the structural advantage of incorporating two K^+^ ions, which act as multidirectional coordination nodes and direct the formation of the extended three‐dimensional architecture observed for **AJE‐2**. The synthetic procedures and characterization data are provided in Section S1 (Supporting Information).

The structures of **AJE‐1** and **AJE‐2** were confirmed by multinuclear NMR spectroscopy, and elemental analysis (Sections S1–S5). Elemental analysis of **AJE‐2** is in good agreement with the calculated values for the anhydrous dipotassium salt.

**FIGURE 2:**
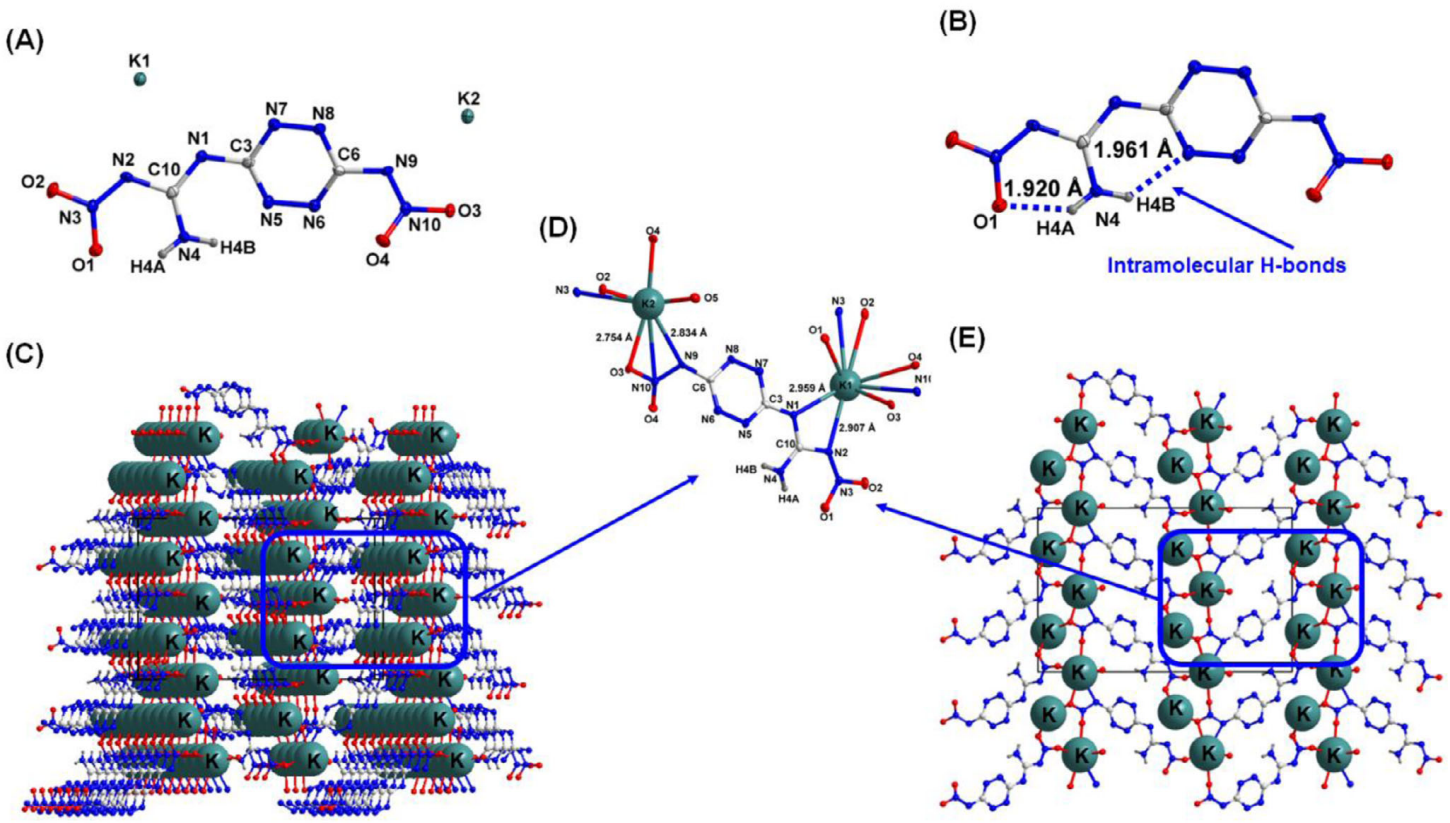
Crystal structure of compound **AJE‐2**. (A) Molecular structure with thermal ellipsoids drawn at the 50% probability level. (B) Intramolecular H‐bond interactions shown as dashed lines in **AJE‐2**. (C) Packing arrangement and coordination network in **AJE‐2**. (D) Coordination of potassium ions with anion. (E) Coordination network in **AJE‐2**.

The single crystals of **AJE‐2** suitable for X‐ray diffraction were obtained from a DMSO/H_2_O solvent system. Single‐crystal X‐ray diffraction analysis shows that **AJE‐2** crystallizes in the orthorhombic space group Pna2_1_ with a calculated density of 1.997 g cm^−3^ at 100 K (Figure 2A). Additional crystallographic details are provided in Figure S1 and Table S1. The single‐crystal structure of **AJE‐2** contains an additional oxygen site bridging neighboring potassium ions. Because no hydrogen atoms associated with this site could be reliably refined, its precise protonation state remains unresolved. The additional site is therefore described conservatively as a bridging oxygen atom rather than being assigned as a lattice water molecule. The asymmetric unit contains one doubly deprotonated tetrazine‐based anion together with two potassium ions. The tetrazine ring remains almost planar, providing a rigid nitrogen‐rich backbone that serves as a bridging platform between adjacent potassium centers.

The nitroimine groups facilitate efficient metal coordination and intermolecular interactions throughout the crystal lattice. The most notable structural feature is the extensive coordination behavior of the potassium cations (Figure 2C).

**K1** is coordinated by both oxygen and nitrogen donor atoms from neighboring anions through multiple K–O and K–N contacts, including K–O distances ranging from 2.725 Å to 3.106 Å and K–N contacts between 2.907 Å and 3.353 Å (Figure 2D). Similarly, **K2** participates in a highly connected coordination environment involving several oxygen atoms of nitro groups together with ring nitrogen atoms, exhibiting K–O distances of 2.639–2.786 Å and K–N distances of 2.834–3.242 Å. These numerous coordination interactions allow each anion to bridge multiple potassium centers and generate an extended coordination network. In addition to metal coordination, the crystal packing is reinforced by intermolecular hydrogen‐bonding interactions involving the amino functionality. Two intramolecular interactions, N4–H4A⋯O1 and N4–H4B⋯N5, stabilize the local conformation of the anion (Figure 2B). These interactions complement the extended K–O/K–N coordination framework generated by the potassium ions. These interactions help stabilize the relative orientations of adjacent anions and complement the coordination framework generated by the potassium ions. The combined effect of K–O/K–N coordination and hydrogen bonding produces a robust 3D framework extending throughout the crystal lattice (Figure 2B–D). The packing diagram shows continuous potassium–anion connectivity that propagates in all directions, generating a densely interconnected network rather than isolated ion pairs. Such cooperative coordination is characteristic of alkali‐metal energetic salts and contributes significantly to the structural integrity (Figure 2D).

From differential scanning calorimetry measurements, exothermic decomposition temperatures of 211°C and 257°C were found for **AJE‐1** and **AJE‐2**, respectively, which indicates a substantial enhancement in thermal stability upon nitration and subsequent formation of the dipotassium framework. Although **AJE‐1** and **AJE‐2** share the same tetrazine‐based backbone, introduction of the additional nitroimine functionality in **AJE‐2** provides new coordination sites that enable binding of a second potassium ion. The 46°C increase is consistent with the greater coordination‐site availability, increased potassium content, and enhanced lattice connectivity of the dipotassium framework. However, because nitration also changes the molecular composition and electronic structure, the improved thermal behavior cannot be attributed exclusively to the crystal architecture. The experimental room‐temperature density of anhydrous **AJE‐2** is 2.01 g cm^−3^ (Table 1) with a calculated enthalpy of formation of 144.4 kJ mol^−1^, which results from the nitrogen‐rich framework. Additional computational details are provided in Section S3. Detonation parameters calculated using the EXPLO5 program [64] based on the experimental density and calculated heat of formation include a detonation velocity of 8093 m s^−1^ and a detonation pressure of 26.4 GPa. These values are provided for comparison with known potassium‐based energetic materials.

**TABLE 1:** Physicochemical and energetic properties of **AJE‐2**.

Compd.	*ρ* ^a^ (g cm^−3^)	Δ*H*_f_ ^b^ (kJ/mol)	Dv^c^ (m/s)	*P* ^d^ (GPa)	*T*_dec_ ^e^ (°C)	IS^f^ (J)	FS^g^ (N)
AJE‐2	2.01^l^	144.4	8093	26.4	257	4	60
LA^h^	4.80	450.1	5920	33.8	315	2.5‐4	0.1‐1
K_2_DNABT^i^	2.11	326.4	8330	31.7	200	1	<1
ICM‐103^j^	1.86	744.7	9111	35.1	160	4	60
TNMT‐K^k^	1.95	146.8	7862	26.3	118	1	5

^a^ Density measured using a gas pycnometer at 25°C.

^b^ Enthalpy of formation.

^c^ Calculated detonation velocity.

^d^ Calculated detonation pressure.

^e^ Temperature of decomposition (onset).

^f^ Impact sensitivity.

^g^ Friction sensitivity.

^h^ Ref. [1].

^i^ Ref. [18].

^j^ Ref. [].

^k^ Ref. [61].

^l^ Density of AJE‐2 measured for anhydrous sample and supported by elemental analysis.

**FIGURE 3:**
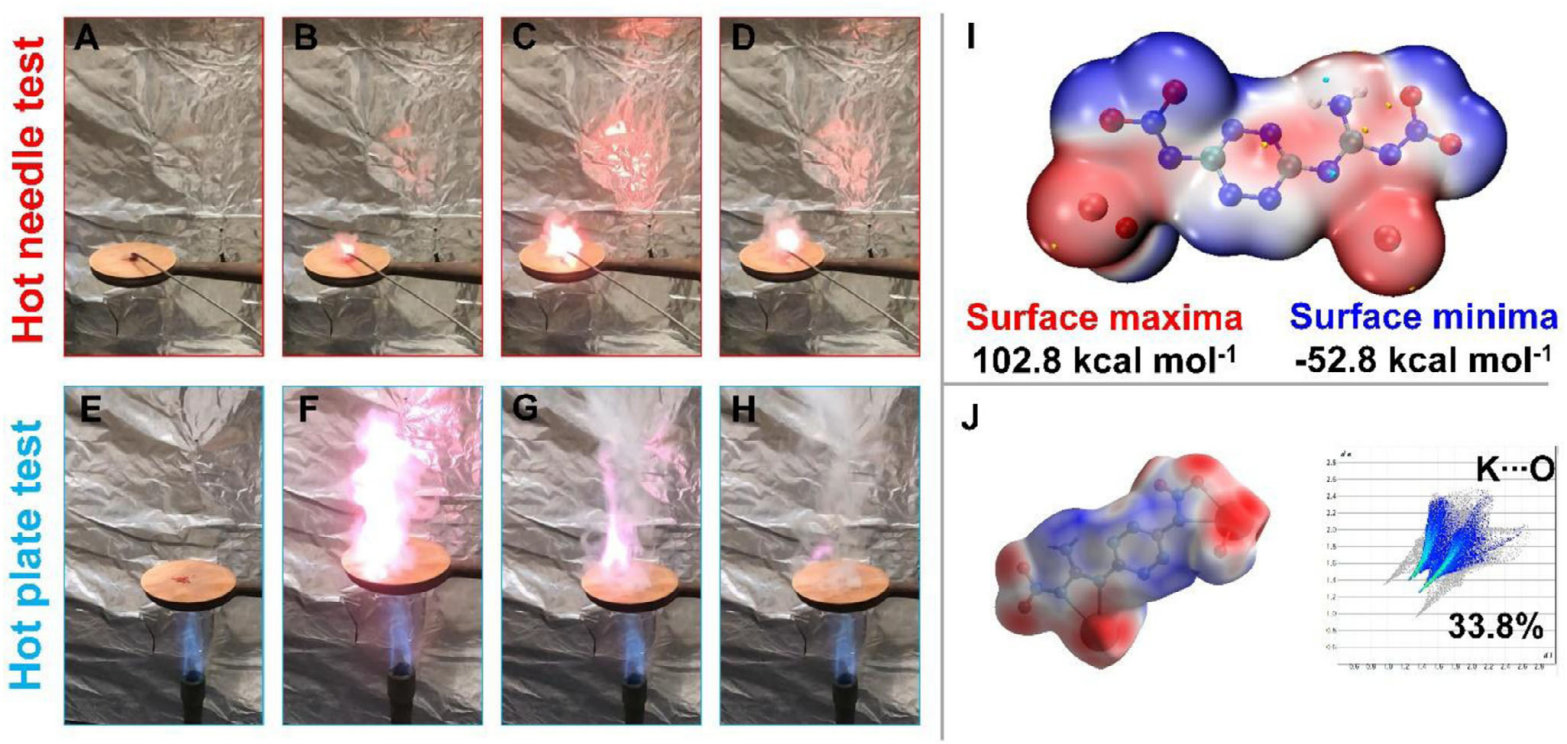
(A–D) Hot needle test of **AJE‐2**. (E–H) Hot plate test of **AJE‐2**. (I) Electrostatic potential (ESP) of **AJE‐2**. (J) Hirshfeld surface analysis and 2D fingerprint analysis of **AJE‐2**.

The impact and friction sensitivities of **AJE‐2** were obtained by using a standard BAM fall hammer and a BAM friction tester, respectively [65, 66]. Sensitivity measurements revealed an impact sensitivity of 4 J and a friction sensitivity of 60 N, confirming that **AJE‐2** can be readily initiated by external mechanical stimuli, similar to known potassium‐based materials. In addition, successful performance in the screening experiments further supports its characteristics similar to known potassium‐based primary energetics. In the hot‐needle experiment, AJE‐2 ignited rapidly with the evolution of dense smoke. In the heated‐plate experiment, high‐speed imaging captured rapid ignition followed by a short‐duration luminous event and violent decomposition (Figure 3A–H).

These experiments are presented only as qualitative thermal‐response screening tests, similar to known potassium‐based materials. Electrostatic potential (ESP) analysis [67] shows pronounced charge separation within the **AJE‐2** anion, with strongly negative regions localized on the nitro oxygen atoms and highly positive regions associated with the protonated nitrogen centers (Figure 3I).

The calculated surface minima and maxima span from −52.8 kcal mol^−1^ to +102.8 kcal mol^−1^. The charge separation reflects the highly polarized nature of the molecule and may contribute to its high sensitivity. Together with the dense potassium‐coordinated framework and high sensitivity, this electronic structure may contribute to the primary behavior of **AJE‐2**. Hirshfeld surface analysis [68, 69] shows that K⋯O interactions constitute the largest contribution to the crystal packing (33.8%), highlighting the dominant role of potassium coordination in the framework assembly centers (Figure 3J). Additional stabilization arises from N⋯H (9.6%) and O⋯H (4.2%) contacts associated with intermolecular hydrogen bonding. Together, these interactions generate a rigid three‐dimensional lattice that contributes to the high density and thermal robustness of **AJE‐2**.

Compared with established primary energetic materials, **AJE‐2** exhibits a favorable combination of density, thermal stability, and energetic performance. Although its sensitivity remains within the range expected for primary explosives, the decomposition temperature of 257°C is notably higher than those of DDNP, ICM‐103, and K_2_DNABT, while approaching that of KDNP. In addition, **AJE‐2** possesses a high room‐temperature density of 2.01 g cm^−3^ and a positive heat of formation, resulting in competitive detonation properties among potassium‐based primary energetic materials. Unlike conventional heavy metal initiators, **AJE‐2** is composed entirely of environmentally benign elements. These properties identify **AJE‐2** as a structurally and energetically notable potassium‐containing material and justify future initiation‐related evaluation.

## 3 Conclusions

In summary, a potassium‐ion‐directed nitrogen‐rich tetrazine framework, **AJE‐2**, was synthesized from a straightforward nitration–deprotonation strategy starting from 1‐(6‐amino‐1,2,4,5‐tetrazin‐3‐yl)‐2‐nitroguanidine. Single‐crystal X‐ray diffraction analysis shows that extensive coordination interactions, together with complementary hydrogen bonds, assemble the dipotassium salt into a robust 3D framework. This 3D organization contributes to the high crystal density and favorable thermal stability observed for **AJE‐2** while preserving the energetic character of the tetrazine‐based scaffold. Energetic evaluation further confirms that **AJE‐2** exhibits the characteristics expected for a potassium‐based energetic material.

## Conflicts of Interest

The authors declare no conflicts of interest.

## Supporting Information

Experimental section, x‐ray crystallography details/crystallographic data, enthalpy of formation, references, and spectral analysis.

## Supporting information




**Supporting File 1**: asia70963‐sup‐0001‐SuppMat.docx

## Data Availability

The data that support the findings of this study are available in the Supporting Information of this article. Crystallographic data have been deposited with the Cambridge Crystallographic Data Centre under CCDC deposition number 2561917.
