# Supplementary material for: Potassium‐Ion‐Directed Three‐Dimensional Assembly of a Dense and Thermally Robust Nitrogen‐Rich Tetrazine Framework
Source: Chem Asian J. 2026 Aug 22;21(16):e70963. doi: 10.1002/asia.70963 (PMC13499537; doi:10.1002/asia.70963)
Supplement: Supplementary file 1 [file ASIA-21-e70963-s001.docx]

**Supporting information**

**Potassium-Ion-Directed Three-Dimensional Assembly of a Dense and Thermally Robust Nitrogen-Rich Tetrazine Framework**

Jatinder Singh,^a^ Parul Saini,^a^ Richard J. Staples,^b^ Jean'ne M. Shreeve^*, a^

^a^ Department of Chemistry, University of Idaho, Moscow, Idaho, 83844-2343, United States.

^b^ Department of Chemistry, Michigan State University, East Lansing, Michigan 48824, United States.

**Corresponding Author**

**Jean’ne M. Shreeve** − Department of Chemistry, University of Idaho, Moscow, Idaho 83844-2343, United States.

Email: jshreeve@uidaho.edu; Fax: (+1) 208-885-5173.

**Table of contents**

| **Section** | **Contents** | **Page No.** |
| --- | --- | --- |
| **S1** | **Experimental section** | **S2** |
| **S2** | **X-ray crystallography details and crystallographic data** | **S4** |
| **S3** | **Enthalpy of formation** | **S6** |
| **S4** | **References** | **S8** |
| **S5** | **Spectral Analysis** | **S9** |

**Section S1. Experimental section**

**Section S1.1. Caution!**

The new compounds are energetic materials which show increased sensitivity toward various stimuli (e.g., higher temperatures, impact, and friction). Proper safety precautions such as leather gloves, face shield, and eye protection must be taken at all times while synthesizing and handling these materials. All materials should be synthesized in milligram amounts.

**Section S1.2. General methods**

All reagents (analytical grade) were purchased from AK Scientific or VWR or Oakwood chemicals and were used as supplied. ^1^H, ^13^C, ^14^N and ^15^N NMR spectra were recorded using a 500 MHz (Bruker AVANCE 500) NMR spectrometer operating at 500.19, 125.78, 36.14, and 50.69 MHz, respectively. Chemical shifts in ^1^H and ^13^C NMR spectra are reported relative to Me_4_Si; ^14^N and ^15^N NMR spectra to MeNO_2_ as an external standard. Abbreviations for multiplicities and descriptors are: s = singlet, br = broad, m = multiplet (denotes complex pattern), and q = quartet. The decomposition points (onset temperature) were obtained using a differential scanning calorimeter (TA Instruments Company, Model: Q2000). Infrared spectra were recorded on a FT-IR spectrometer (Thermo Nicolet 6700) equipped with an ATR assembly. The densities were measured at ambient temperatures by employing a gas pycnometer (Micromeritics AccuPyc II 1340). The impact and friction sensitivities were determined by using a standard BAM drop hammer and BAM friction tester. Elemental analyses were carried out on a Vario Micro cube Elementar Analyser.

**Section S1.3. Synthesis of compounds**

Precursor P1, P2, and P3 were synthesized following the literature procedure.^[1]-[2]^

**Synthesis of AJE-1.** Precursor P2 (1.00 g, 5.02 mmol) was suspended in ethanol (30 mL), and a solution of KOH (0.31 g, 5.52 mmol) in ethanol was added dropwise at room temperature. The resulting mixture was stirred for 1 h at room temperature. The resulting red solid was collected by filtration, washed with ethanol (2 × 10 mL), and dried under reduced pressure to give **AJE-1** as a red solid. Yield: 92%; T_d_ = 211 °C. ^1^H NMR (500 MHz, d_6_-DMSO): 8.28 (bs, 2H), 6.98 (bs, 2H); ^13^C NMR (125 MHz, d_6_-DMSO): 164.8, 160.2, 159.9; Elemental analysis: Calcd for C_3_H_4_KN_9_O_2_.H_2_O: C, 14.12; H, 2.37; N, 49.39. Found: C, 14.13; H, 2.35; N, 49.48.

**Synthesis of compound AJE-2**. Precursor **P2** (1.00 g, 5.02 mmol) was added portion wise to 100% nitric acid at 0 °C, and the reaction mixture was stirred at the same temperature for 15 min. Excess nitric acid was removed under reduced pressure, and the resulting solid **P3** was suspended in ethanol (30 mL). A solution of KOH (0.59 g, 10.5 mmol) in ethanol was then added dropwise at room temperature. The reaction mixture was stirred for 1 h, after which the resulting red solid was collected by filtration, washed with ethanol (2 × 10 mL), and dried under reduced pressure to give **AJE-2** as a red solid. Yield: 90% (starting from P3); T_d_ = 257 °C. ^1^H NMR (500 MHz, d_6_-DMSO): 8.80 (bs, 2H);

^13^C NMR (125 MHz, d_6_-DMSO): 164.9, 162.1, 161.4; Elemental analysis: Calcd for C_3_H_2_K_2_N_10_O_4_: C, 11.25; H, 0.63; N, 43.73. Found: C, 11.32; H, 0.89; N, 43.45.

**Section S2.** **X-ray crystallographic details and crystallographic data**

**Section S2.1. Sample preparation**

Single crystals of compounds **AJE-2** suitable for single-crystal X-ray analysis were obtained from DMSO:H_2_O.

**Section S2.2. Data collection**

Crystals with suitable dimensions were mounted on a nylon loop with Paratone oil. Data were collected using a XtaLAB Synergy, Dualflex, HyPix diffractometer equipped with an Oxford Cryosystems low-temperature device, operating at T = 99.9(4) K. The structures were solved with the ShelXT^[3]^ solution program using dual methods and by using Olex2.^[4]^ The model was refined with ShelXL^[5]^ using full matrix least squares minimization on *F*^2^. The thermal ellipsoids and packing diagrams of X-ray structures in the main article and supplementary material are plotted using Diamond 3.2 software.

**Section S2.3. Crystal structures and crystallographic data.**

**Figure S1:** Thermal ellipsoid plot for compound **AJE-2**. Thermal ellipsoids are drawn at 50% probability level. Data collected at 100 K. CCDC # 2561917.

**Table S2:** Crystallographic data for **AJE-2**.

| **Compound** | **AJE-2** |
| --- | --- |
| CCDC | 2561917 |
| Formula | C_3_H_2_K_2_N_10_O_5_ |
| *D_calc._*/ g cm^-3^ | 1.997 |
| *m*/mm^-1^ | 7.980 |
| Formula Weight | 336.35 |
| Colour | red |
| Shape | plate-shaped |
| Size/mm | 0.27×0.14×0.04 |
| *T*/K | 100.0(3) |
| Crystal System | orthorhombic |
| Flack Parameter | -0.014(19) |
| Hooft Parameter | 0.007(11) |
| Space Group | *Pna*2_1_ |
| *a*/Å | 13.7795(3) |
| *b*/Å | 3.79465(9) |
| *c*/Å | 21.3972(4) |
| *a*/^°^ | 90 |
| *b*/^°^ | 90 |
| *g*/^°^ | 90 |
| V/Å^3^ | 1118.83(4) |
| *Z* | 4 |
| *Z'* | 1 |
| Wavelength/Å | 1.54184 |
| Radiation type | Cu K*_a_* |
| *Q_min_*/^°^ | 4.132 |
| *Q_max_*/^°^ | 79.851 |
| Index range h | -16 ≤ h ≤ 17 |
| Index range k | -4 ≤ k ≤ 2 |
| Index range l | -26 ≤ l ≤ 27 |
| Measured Refl's. | 4098 |
| Indep't Refl's | 1787 |
| Refl's I≥2*s*(I) | 1720 |
| *R*_int_ | 0.0425 |
| Parameters | 186 |
| Restraints | 1 |
| Largest Peak/eÅ^3^ | 0.612 |
| Deepest Hole/eÅ^3^ | -0.501 |
| GooF | 1.059 |
| *R_1_* (I≥2*s*(I) / all) | 0.0478 / 0.0488 |
| *wR_2_* (I≥2*s*(I) / all) | 0.1297 / 0.1311 |

**Section S3. Enthalpy of formation**

**Section S3.1. Isodesmic reactions**

The **∆H_f_** (enthalpies of formation) for new compounds were calculated by using an isodesmic reaction (Scheme S1).

**Scheme S1**: Isodesmic reactions.

The single crystal structure was used for geometric optimization and frequency analyses using the B3LYP functional with the 6-31+G** basis set. The single-point energies were obtained at the MP2/6-311++G** level.^[6]^

| Compound | ZPE | H_corr_ | scaled ZPE | ∆H_T_ | Mp-6-311++g** | Corrected | ∆H_r_ | ∆H_r_ (kJ/mol) | ∆H_f_ (kJ/mol) |
| --- | --- | --- | --- | --- | --- | --- | --- | --- | --- |
| **Dianion re** | **0.102156** | **0.116898** | **0.09807** | **0.014742** | **-961.580348** | -961.46754 | **0.08621656** | 226.3615566 | **515.6884434** |

**Dianion** (MP2 = **-961.580348**)

Number of imaginary frequencies (NImag) = 0

The atomization energies for cations were calculated by using the G^2^*ab* *initio* method ^[7]^ All of the optimized structures were characterized to be true local energy minima on the potential energy surface without imaginary frequencies. In case of the energetic salts, the solid-phase heats of formation were obtained based on a Born–Haber energy cycle.^[8]^

**Section S4. References**

[1] H. Wei, H. Gao, J. M. Shreeve, *Chem. - A Eur. J.* **2015**, *21*, 2726–2727.
[2] J. Singh, R. J. Staples, J. M. Shreeve, *Mater. Horizons* **2026**, <https://doi.org/10.1039/d6mh00935b>.
[3] G. M. Sheldrick, *Acta Crystallogr. Sect. A Found. Crystallogr.* **2008**, *64*, 112–122.
[4] G. M. Sheldrick, *Acta Crystallogr. Sect. C Struct. Chem.* **2015**, *71*, 3–8.
[5] V. Dolomanov, L. J. Bourhis, R. J. Gildea, J. A. K. Howard, H. Puschmann, *J. Appl. Crystallogr.* **2009**, *42*, 339–341.
[6] O. Suleimenov, T.-K. Ha, *Chem. Phys. Lett.* **1998**, *290*, 451–457.
[7] R. G. Parr, Y. Weitao, *Density-Functional Theory of Atoms and Molecules*, Oxford University Press, **1995**.
[8] H. D. B. Jenkins, D. Tudela, L. Glasser, *Inorg. Chem.* **2002**, *41*, 2364–2367.

**Section S5. Spectral Analysis**

**Figure S2**: ^1^H NMR spectrum of **AJE-2** in DMSO-d6.

**Figure S3**: ^13^C NMR spectrum of **AJE-2** in DMSO-d6.

**Figure S4**: ^14^N NMR spectrum of **AJE-2** in DMSO-d6.

**Figure S5**: DSC of **AJE-2** at the heating rate of 5°C/min.
